# Long-Range UHF RFID Tag for Automotive License Plate

**DOI:** 10.3390/s21072521

**Published:** 2021-04-04

**Authors:** Youchung Chung, Teklebrhan H. Berhe

**Affiliations:** Information and Communication Engineering Department, Daegu University, Kyungsan 38453, Korea; tek.hintsa@gmail.com

**Keywords:** automotive, license plate tag, UHF RFID tag, long range RFID tag, back-lobe RFID tag

## Abstract

In this paper, various locations of an Ultra High Frequency (UHF) Radio Frequency Identification (RFID) tag on automotive license plates have been considered based on the radiation pattern of the tag antenna. A small, 130 × 50 mm, passive loop antenna type UHF RFID tag for an automotive license plate was simulated with an EM simulation CST program. It was designed to have a larger back-lobe radiation pattern since the front side of the tag faces the back side of the plate holder to protect the tag antenna from bugs and dust when the automobile runs. The tag was attached to the side of a license plate holder with a dimension of 520 × 110 mm, the typical size of the standard license plate. The reflection coefficient of the tag antenna is −21 dB at 920 MHz, and the gain of the tag antenna is 6.29 dBi at the back-lobe. The reading range of the tag antenna with the plate holder, which was measured in an open field, is about 10.3 m, and the reading range of the tag installed on the bumper from the front of the vehicle is 9.4 m. The tag antenna is small enough to apply to a real automobile, and it is applicable because it uses the back-lobe pattern, so it does not require an extra device for protection from damage.

## 1. Introduction

Radio Frequency Identification (RFID) is used for the recognition of objects or people. RFID technology is a system that recognizes information from a reflected signal, and it is widely used as an application to find out information about an object [1]. These technologies are widely used in animal management and also for traceability in sectors such as automobiles, pharmaceuticals, precious metal management, steel distribution and logistics, and the high-end liquor industry by tracking and managing distribution logistics [1,2,3].

Currently, RFID systems of various frequency bands are used, and the application fields of RFID are classified according to the frequency bands. Low Frequency (LF) 125~135 kHz and High Frequency (HF) 13.56 MHz bands are used for short-distance use, and the active Ultra High Frequency (UHF) 433 MHz system is used for long-distance applications such as container management. The UHF RFID band, 860~960 MHz, is generally used for logistics management, and the 2.45 GHz band is used for check authentication and passport recognition. A UHF RFID system operates at bands of 902~928 MHz for North America, 865~ 867 MHz for Europe, 916.7~923.5 MHz for Japan, and 917~923.5 MHz for Korea [1,3].

International standards for each frequency band are stipulated by the International Standard Organization (ISO) and the International Electro-Technical Commission (IEC) as follows: ISO 18000-2 defines the band below 135 kHz, and ISO 18000-3, 13.56 MHz, specifies smart cards and wireless payment. ISO 18000-7 specifies the active 433 MHz RFID system, and ISO 18000-6 specifies the 860 to 960 MHz UHF band. The 2.45 GHz band is specified by ISO 18000-4 [4,5,6,7]. Recently, ISO 18000-6C and 6D were added to the ISO standard, and 18000-61~18000-64 were specified in the ISO 18000-6:2013 document [8].

The UHF RFID tag is used to recognize objects and for tracking valuable or expensive items. It is also used for tracking cars, accessing control of automobiles in a parking lot, and crossing border control [9,10,11,12]. Since UHF RFID tags can be recognized over long distances, they are attached to car windshields and used for toll highway cost settlement as well as by law enforcement to determine driver eligibility and stop line enforcement [13,14,15]. Vehicle registration is usually done by attaching a tape-type registration to the front or rear license plate to prove that the vehicle is legally registered. If the UHF RFID tag is installed on a license plate or license plate holder, owners of the automobile do not need to attach a separate registration certificate on the plates or bumpers since it can be automatically recognized as a legally registered automobile at an RFID gate.

The electronic plate antennas and tag designs for license plates have been researched for many years. The performance of the proposed tag antenna is closely related to the type of material of the license plate and the material near the license plate. The microstrip patch tag antenna [16], microstrip dipole UHF tag antenna, and a loop type tag antenna [17] have all been placed behind non-metal plates. Since they have been designed for non-metal license plates, the tag antenna installed behind a metal license plate will not operate properly as a tag antenna. A slot-type antenna is proposed to solve this problem with metallic objects [18,19], but the current license plate design should be changed. In reference [20], an active 2.45GHz RFID system for a license plate with a meander slot antenna was reported. Active slot-type dipole 2.45 GHz and 433MHz active tag antennas for the license plates have been introduced [20,21]. 

The drawback with this active RFID plate is that it is expensive compared to a passive tag since it requires a power source and digital communication system. In order to improve the identification accuracy, a dual resonant frequency diversity design was conducted in article [21]. This license plate tag antenna has the dual resonant frequency of an active band (433 MHz) and also a passive UHF RFID band to reduce identification error. A study on identifying an illegal automobile detection system using a UHF RFID tag antenna for a seal-bolt shape was reported in [22]. A very small UHF RFID band tag antenna, the size of an American quarter coin, was designed and applied to the rear plate license plate fixing seal-bolt and implemented a system to recognize illegal vehicles. The reading range of this seal-bolt-type UHF tag was 3.5 m since the size of the seal-bolt tag was small. Reference [23] presents the design of an RFID tag antenna using the vehicles’ side-view mirror. This study proposed the position of tag antennas for vehicles. In order to control dipole tag antenna directivity in the side-view mirror of a vehicle, research was done to find the optimum location, but the size of the tag was limited.

Automobile license plate tags are adhered to or mounted on the license plate on the front of the vehicle, and the tags are read when the car is moving towards the RFID reader antenna. Since every vehicle has a license plate, the available plate structure is used to design the tag antenna. The current tag antennas in the market have a short reading range of around 5~8 m, and an active RFID system is expensive. Therefore, a long-range passive UHF RFID tag antenna for vehicle license plates is required. 

This article presents the design of a passive Ultra High Frequency (UHF) Radio Frequency Identification (RFID) tag antenna that is applied to the vehicular license plate and attached to the vehicle bumper [24]. To improve the identification ratio and time, the tag antenna should have a frontal beam pattern. Therefore, a small size passive UHF RFID tag antenna, 130 × 50 mm, for the license plate was designed, which faces the bumper rather than the front. The tag has a larger back-lobe radiation pattern since the front side of the tag faces the bumper side to protect the tag antenna from bugs and dust when the automobile runs. In Section 2, the general procedure and matching method have been reviewed. The shape of the UHF RFID antenna, the performance of the tag antenna, and the proper location of the tag antenna on the plate based on the beam pattern have been considered in Section 3. In Section 4, there is a conclusion to this article.

## 2. RFID Tag Antenna Design Process

To design a UHF RFID tag antenna, the impedance (*Za*) of a tag antenna should be the complex conjugate of the impedance of the RFID integrated circuit (IC) of the tag (*Zc*). Thus, *Za* = Z_C_^*^, and is separated into real and imaginary parts. We have designated the real part *Ra = Rc* and the imaginary part *Xa = −Xc.* Since the imaginary part of IC chip impedance is about −120 *j*, to achieve the high inductive impedance of the tag antenna, the T-matching structure is used. 

The total power received by the tag is defined by Friss Equation (1) [3,25,26]. The parameters involved in the transmitting and receiving system are

(1) $$P_{r}=P_{t}{\left(\frac{\lambda }{4\pi R}\right)}^{2}G_{t}G_{r}$$

where, the parameters are *Pt* (transmitted power), *Gt* (transmission antenna gain), *Pr* (received power), *Gr* (gain of tag antenna), and wavelength 𝝀 and *R* distance between the transmitting and receiving tag antennas.

The read-range is calculated with Equations (2) and (3). Equation (2) shows the matching coefficient τ, which determines how well the impedance of a tag antenna and IC chip are matched to the chip. The reading range is calculated with Equation (3) [3,26]. The threshold power *P_th_* of an Alien Higgs-4 chip equals to −20.5 dBm, as provided by the manufacturer.

(2) $$\tau \;=\;\frac{4R_{a}^{2}}{|Z_{a}+Z_{C}{|}^{2}}$$

(3) $$R=\;\frac{\lambda }{4\pi }\sqrt{\;\frac{P_{t}G_{t}G_{r}}{P_{th}}\;\tau }\;0\;\leq \;\tau \;\leq \;1,$$

## 3. Design of UHF RFID Tag Antenna for a License Plate

**Figure 1:**
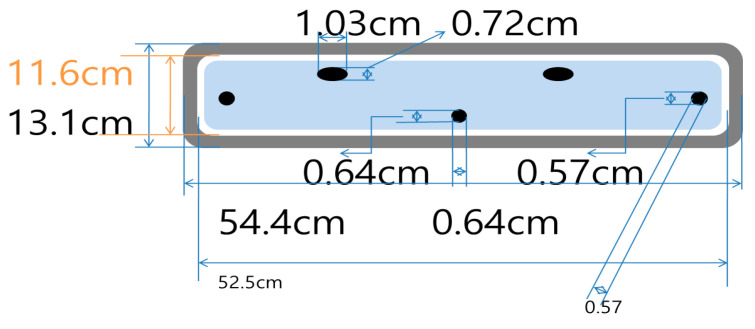
Size of typical license plate holder.

There are two commonly used license plates, with the sizes of 33.5 × 15.5 cm and 52 × 11 cm. The second size of 52 × 11 cm was used for the development process since it is more popular around the world. The metal plate holder shown in Figure 1 is the typical size of a standard license plate (52 × 11 cm) used in many counties. The plate had two holes at the top of the holder to the bumper and two holes at the sides of the holder. They are used for mounting the license plate and the holder to the bumper. 

A λ long loop antenna structure was used for the UHF 920 MHz RFID tag antenna to have a frontal radiation pattern toward the front of the vehicle. The T-matching method was used to match the input impedance of the tag antenna to the conjugate of the impedance of the RFID chip. Because of the impedance change according to the materials near the car and the effect of bolts and nuts, the material near the tag antenna should be considered during the design process. The beam pattern should be considered since the tag around the plate or on the bumper should be identified by a reader antenna installed on a gate on the road.

**Figure 2:**
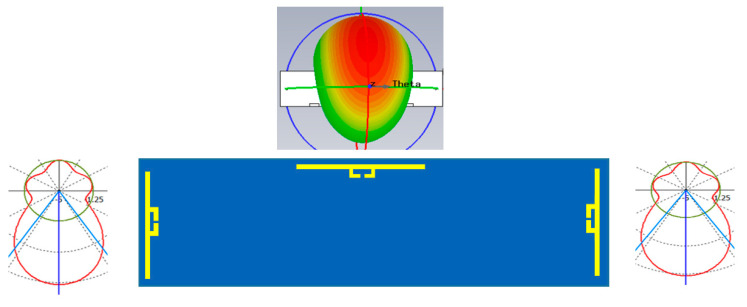
The possible locations of tag antenna installed around the license plate.

Since there is a metal license plate in front of the plate holder, the tag cannot be installed at the center of the plate holder. The simplest possible installation locations of the dipole type tag antennas are on the top and the side of the license plate, and the radiation of patterns of those possible antennas are displayed and considered in Figure 2. Due to the metal plate and plate holder, the radiation patterns of the dipoles installed on the sides are pointed towards the bumper side, and the radiation pattern of the dipole installed on the top side is tilted 30 degrees towards the bumper side.

As mentioned in Section 1, the tag should have a frontal beam pattern since it improves the recognition rate and time at the gate on the road when the automobile passes through the gate. Therefore, the dipole shape of the tag antenna is not proper and the flat antenna structure with a frontal beam pattern may be suitable for this application. Even though a flat patch antenna has a frontal pattern, it is hard to achieve the T matching structure, and it should be faced toward the front side, which can be vulnerable to dust and damage when the automobile runs. 

**Figure 3:**
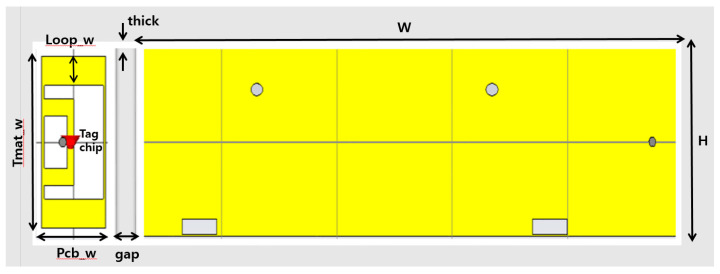
License plate tag antenna attached next to the plate holder facing bumper side.

**Figure 4:**
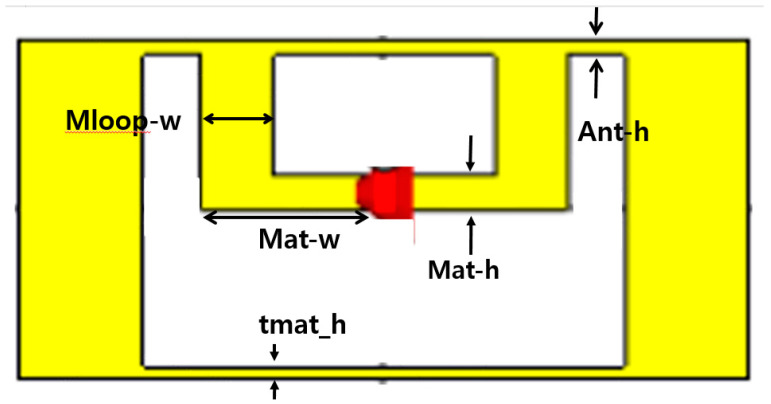
Parameters of the Radio Frequency Identification (RFID) tag antenna in Figure 3 enlarged and rotated clockwise 90 degrees.

Figure 3 and Figure 4 show the structure of the proposed automobile plate for a plastic vehicle bumper and tag antenna. It is composed of a metal plate as a license plate and plate holder, bolts and nuts, and a part of the vehicle bumper. The bumper is made of plastic with a dielectric constant of ε_r_ = 3.2. In this design, the tag antenna is designed to attach to a metal part of the license plate holder. Figure 3 shows the proposed tag antenna attached next to the standard plate holder, which was shown in Figure 1. The loop shape tag antenna facing the bumper is placed on the side of the license plate to have the frontal pattern. The tag antenna has a rectangular shape with a dimension of 130 × 50 mm and is attached next to the license plate holder as shown in Figure 3. The tag facing the bumper side in Figure 3 is enlarged and drawn in Figure 4 with the detailed parameters of the antenna. This tag antenna uses the back-lobe pattern to be recognized by readers since it faces the bumper side.

The proposed loop antenna was simulated using CST simulation software. A discrete port was used to represent the tag terminal. There was a gap of 2 mm between the two edges of the discrete port for the RFID strap. The simulated results of the proposed antenna gave acceptable performances at a center frequency of 920 MHz for UHF RFID applications. To protect the tag antenna, the front side of the tag antenna faces the bumper side because many materials, like bugs and dust, hit the bumper when the vehicle runs. The dielectric constant of the RF IS-680 board for the UHF RFID tag antenna fabrication was 3.2, and the thickness of the IS-680 board was 30 mil.

**Figure 5:**
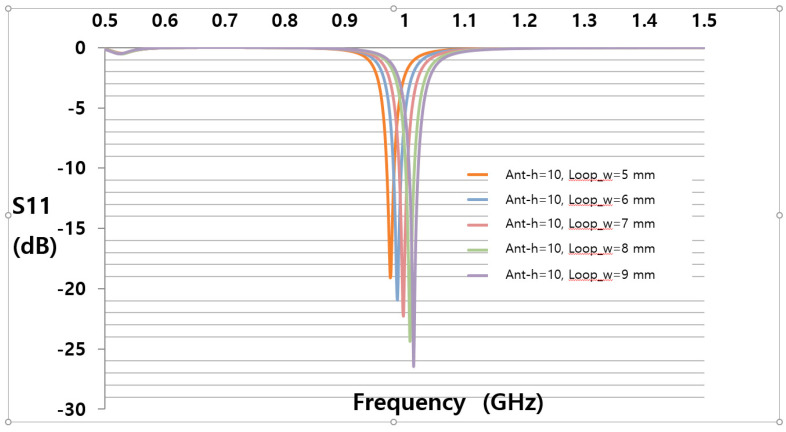
Simulation results of the parameter sweeps.

**Figure 6:**
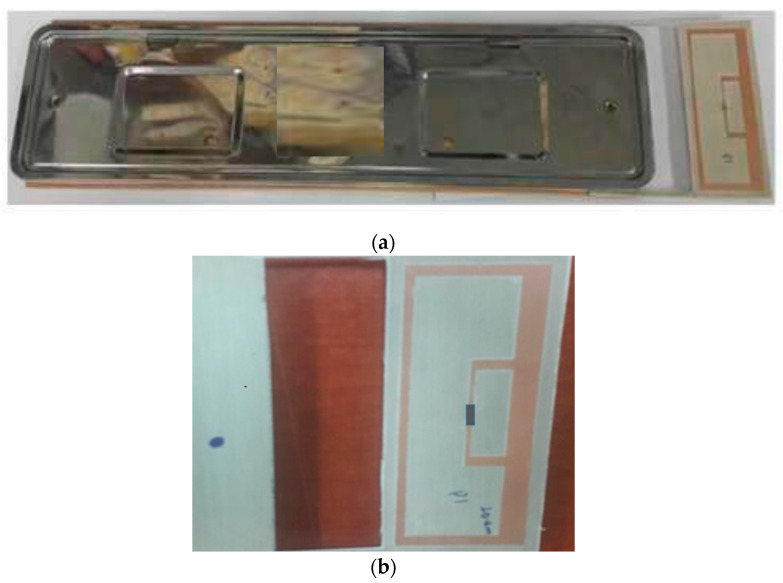
(**a**) Fabricated tag antenna with the plate holder (bumper side). (**b**) Fabricated prototype tag antenna.

This plate tag antenna structure consists of several parameters with the difference in width and length of each element. The most dominant parameters in simulation results are Loop_w and Ant_h. In order to achieve optimum value during the simulation stage of the proposed antenna, parametric studies were conducted. Parameter sweeps were used for the optimization of the parameter elements. Figure 5 shows the simulation result using parametric sweeping with the center frequency of 920 MHz. From the simulation result study, the optimal parameter values occurred at Loop_w = 5 and Ant_h = 10 mm. At the operating frequency of 920 MHz, the simulated value of the reflection coefficient was −29.55 dB. The optimized parameters of the tag antenna for having a larger back-lobe pattern are shown in Table 1. The fabricated tag antenna is shown in Figure 6a,b and has a hole on the left side of the tag to mount on the right side of the plate holder.

**Figure 7:**
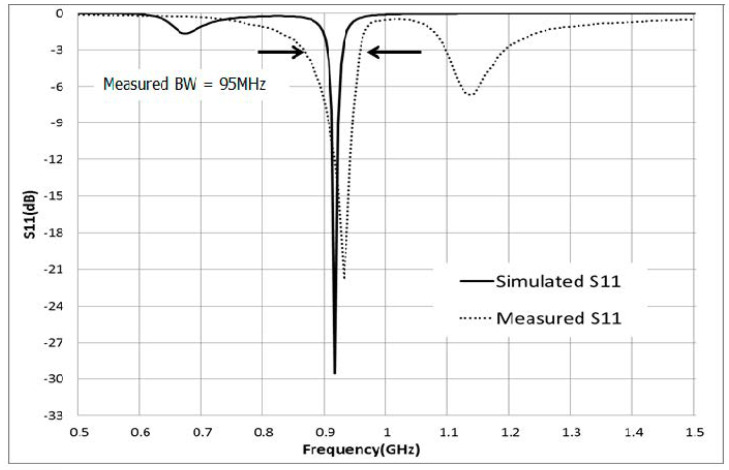
Simulated and measured S11 of tag antenna.

Figure 6a shows the fabricated tag antenna with the plate holder. The tag is on the right side of the holder. The larger picture of the tag is shown in Figure 6b with a mounting hole. The dielectric constant of 3.2 and a thickness of 30 mil RF IS-680 board has was used for fabrication of the tag antenna. The UHF Higgs-4 RFID strap was bonded to the tag antenna. An Alien Higgs-4 chip has *R* = 1800 kΩ and *C* = 0.95 pF. The frequency is ω = 2 πf, where f is 920 MHz. The chip impedance is *Z_c_* = 18.258 − j180.32, and the simulated and measured S11 are shown in Figure 7. Results of the simulation and measurements show that the proposed tag antenna has a reflection coefficient of 29.55 dB and 17 dB, respectively, at 920 MHz. Measurement was done with the Agilent N5230 PNA network analyzer.

**Figure 8:**
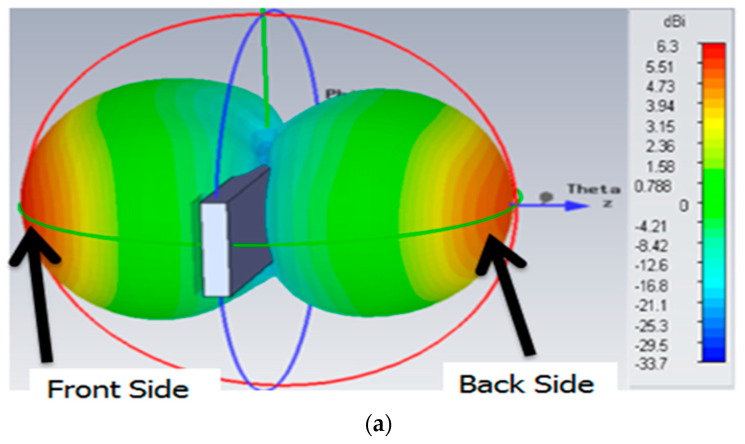

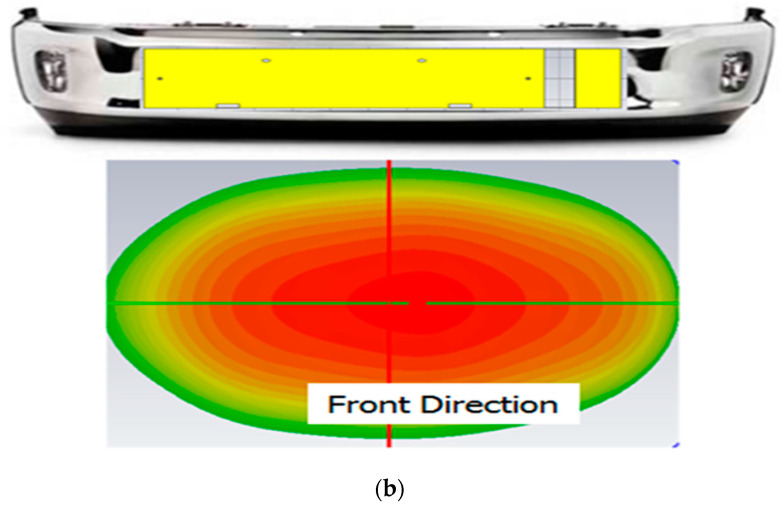
(**a**) Side view of 3D radiation pattern with plate holder and at 920 MHz. (**b**) Front view of radiation pattern of the tag antenna at 920 MHz.

**Figure 9:**
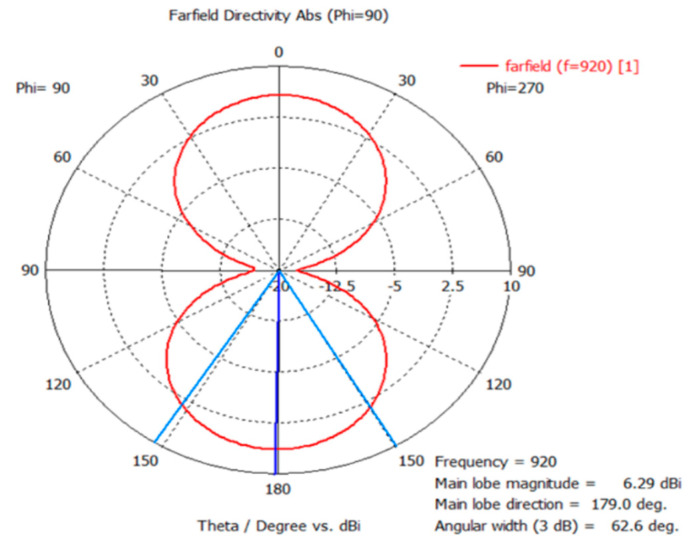
Two-dimensional radiation pattern of the tag antenna at 920 MHz.

The 3D radiation pattern was simulated with the plastic material of the bumper since any metallic material further than 2 cm away from the tag does not change the reading range of the tag. Therefore, the simulated pattern of the tag shown in Figure 8a shows that there was a strong signal radiating from the plate towards the front and back side of the road, where readers were deployed. Figure 8b shows the front view of the radiation pattern of the tag antenna and shows the center of the pattern is little bit tilted towards the tag side. The gain of the main beam is 6.3 dBi. Since this is a loop antenna, the front-and back-lobe of beam patterns are almost identical. The materials near the tag antenna can affect the characteristics of the tag antenna. Therefore, all these conditions were included in our numerical simulation. The 3D radiation pattern gives a good illustration of the radiated field distribution as compared to the 2D pattern in Figure 9.

The 3D radiation pattern in Figure 8 shows that there was a strong signal radiating from the plate towards the front and back side of the road, where readers were deployed. 

**Figure 10:**
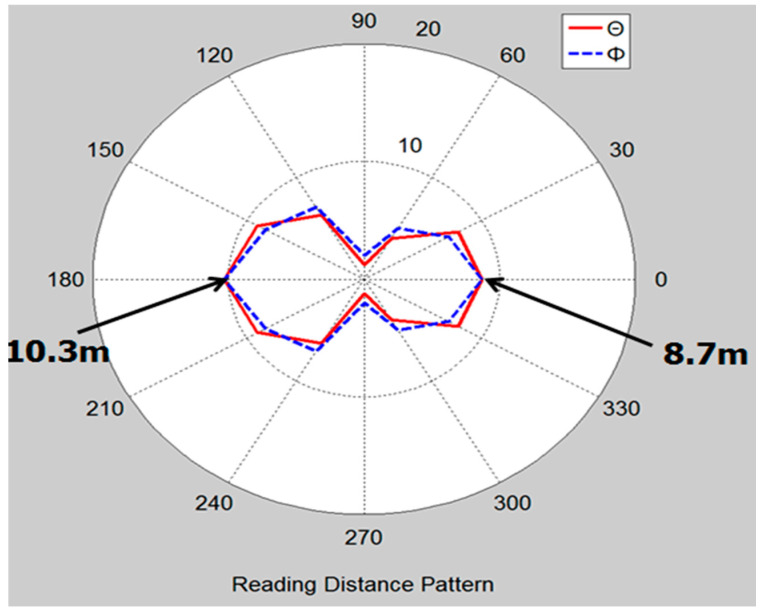
Measured reading range pattern with the plate holder.

Figure 10 shows the measured reading range pattern of the fabricated license plate tag antenna with the holder, according to theta and phi angles using a linear polarization (LP) antenna. Since the antenna is optimized for having a larger back-lobe pattern, the back side pattern is a little bit larger. An angle of 0 degrees implies the front of the tag antenna on the bumper side, and an angle of 180 degrees indicates the front of the car, which is the back side of the tag antenna. Therefore, while measuring the reading range of a tag antenna attached to the car with the bumper, the back-lobe, 180 degrees, is our desired signal. 

The reading ranges of the tag antenna with the plate holder were 8.7 m at 0 degrees and 10.3 m at 180 degrees. The reading range of the tag antenna installed on the bumper was 9.4 m from the front of vehicle and could not be read on the back side of vehicle. From the reading range pattern in Figure 10, we can see that the license plate tag antenna has a long reading distance when the tag antenna is at 180 degrees with respect to the reader antenna.

## 4. Conclusions

In this article, a design for a small 130 × 50 mm UHF RFID passive tag for automotive license plates has been described. The parameters of the tag antenna were optimized to have a larger back-lobe pattern because the front side of the tag faces the bumper side to protect from bugs and dust when an automobile is driven. The prototype tag was attached to the side of a license plate holder with the dimensions of 520 × 110 mm. The simulation included material of the bumper around the tag antenna as described in Section 3. The reading range of the tag itself was 10.4 m, and the reading range of the tag attached on the front bumper of the vehicle was 9.4 m. The tag antenna is small enough to apply to real automobiles and is applicable since it uses the back-lobe pattern, so it does not require extra protection from damage.

There is more work to be done—for example, the size of the tag could be smaller with better performance, and the back-lobe-pattern could be larger than the current design by using other structures of the antenna and through further optimization. The tag could also be designed and inserted into the bumper structure. 

## Figures and Tables

**Table 1:** Optimized tag antenna parameters.

Parameters.	Size (mm)	Parameters	Size (mm)
Pcb_w	50	Mat-w	25
H	130	Mat-h	2.2
W	525	Loop_w	5
tmat_h	4	Mloop-w	5
Ant-h	10	gap	40
Tmat_w	125	thick	5

## Data Availability

Not applicable.
